# Unprecedentedly high global forest disturbance due to fire in 2023 and 2024

**DOI:** 10.1073/pnas.2505418122

**Published:** 2025-07-21

**Authors:** Peter Potapov, Alexandra Tyukavina, Svetlana Turubanova, Matthew C. Hansen, Louis Giglio, Andres Hernandez-Serna, André Lima, Nancy Harris, Fred Stolle

**Affiliations:** ^a^Department of Geographical Sciences, University of Maryland, College Park, MD 20740; ^b^World Resources Institute, Washington, DC 20002

**Keywords:** forest change, forest fires, active fires, climate change

## Abstract

Global forests provide key ecosystem services, from climate regulation to biodiversity habitat, but are under increasing pressure from the combined impacts of climate and land use change. Here, we show that forest disturbance due to fire is growing globally, with the most dramatic increases in intact forest landscapes, highlighting an existential threat to remaining high biomass, high biodiversity forests. The global annual area of forest disturbance due to fire for 2023 and 2024 was highest since the beginning of monitoring in 2001. Compared to 2002–2022 average annual forest disturbance due to fire, the 2023–2024 average was 2.2 times higher globally and 3 times higher in the Tropics. More than ¼ of all 2024 forest disturbance from fire occurred in tropical forests. We found a statistically significant increasing trend of forest disturbance due to fire from 2002 to 2024 in all climate domains except Subtropical. High forest, low deforestation tropical countries were not exempt, with Guyana and the Republic of the Congo experiencing record forest disturbance due to fire. Our results agree with recently estimated increases in global forest fire emissions and active fire detections. The unprecedented scale of fires in the world’s most remote forests is a potential harbinger of ecosystem tipping points. Protecting these remaining unfragmented high conservation value forests from this threat poses a daunting and as yet undeveloped policy and capacity challenge.

## Results

**Fig. 1.**
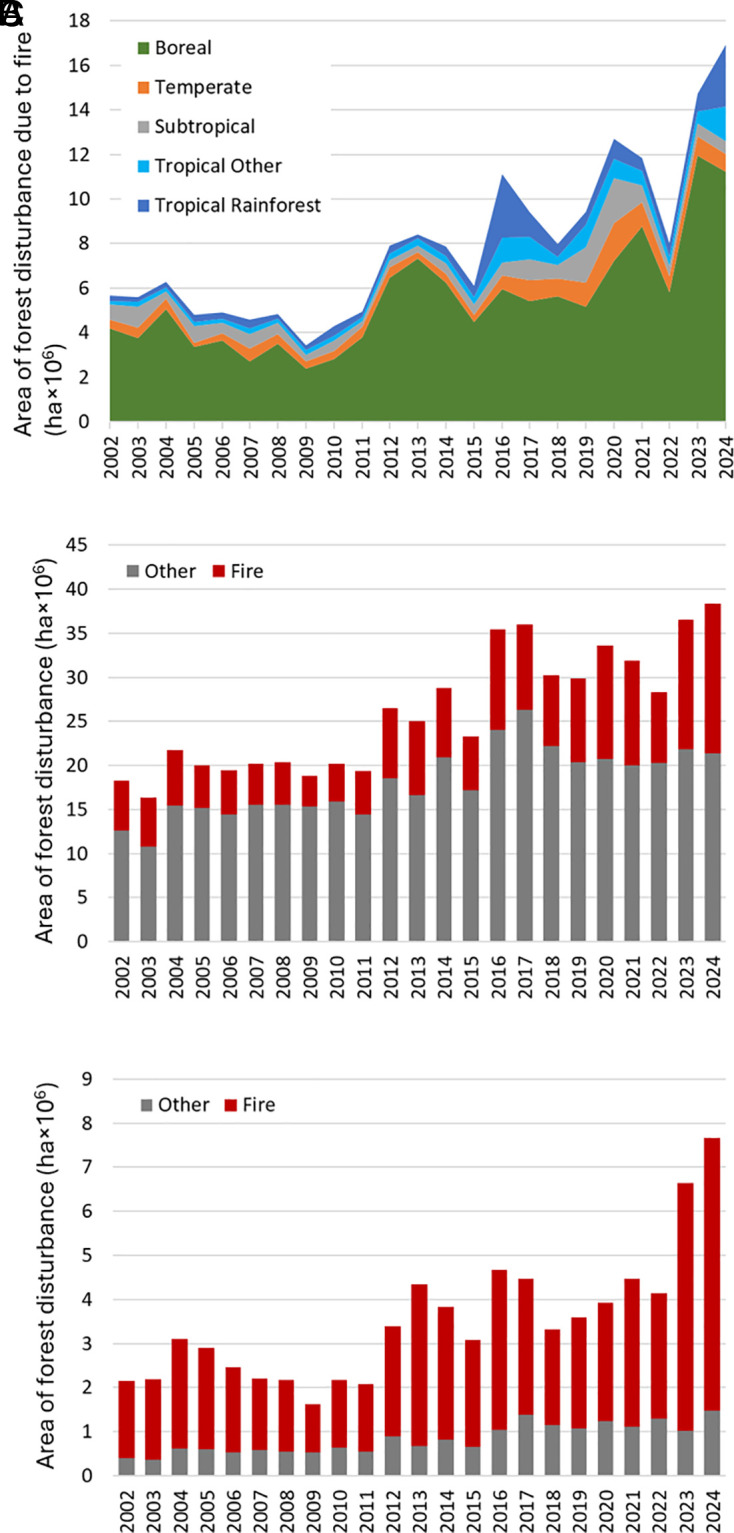
(*A*) Annual area of forest disturbance due to fire by climate domain globally, 2002 to 2024. Annual area of forest disturbance due to fire and other proximate causes within (*B*) all forests and (*C*) Intact Forest Landscapes (IFL), 2002 to 2024.

The 2023 and 2024 annual global forest disturbance area reported by the University of Maryland (1, 2) was highest since the observation began in 2001 (36.6 and 38.3 million ha, respectively). Of the total 2023 and 2024 forest disturbance area, 42% was due to forest fires, which is a much higher percentage than the 26 to 29% estimated earlier for the 2001–2019 interval (3). A statistically significant (Mann–Kendall test, *P* < 0.05) increasing trend in forest disturbance due to fire from 2002 to 2024 was confirmed globally and for all climate domains except Subtropical (Fig. 1*A*). Comparing the 2023 to 2024 average annual forest disturbance due to fire with the 2002 to 2022 average, we found that it increased 2.2 times globally; the largest increase was found in tropical (3 times higher) and boreal (2.3 times higher) forests. During two extreme fire years, 2016 and 2024, more than ¼ of all forest disturbance due to fire occurred in tropical forests. The observed increase in annual forest disturbance due to fire over the past 24 y is consistent with earlier reports of burned forest area increase in all forests [2001 to 2019, (3)] and closed-canopy forests [1998 to 2015, (4)].

At the continental scale, the largest increase in average area of forest disturbance due to fire between 2023–2024 and 2002–2022 intervals was in North America (3.7 times), Latin America (3.4 times), and Africa (2.4 times). The statistically significant increasing trend in fire-related forest disturbance was confirmed for Africa, Eurasia, Latin America (Mann–Kendall test, *P* < 0.05), and North America (*P* < 0.1). The top ten countries with the largest annual average forest disturbance due to fire increase between 2002–2022 and 2023–2024 intervals include Canada, Russia, Brazil, Bolivia, Mexico, Democratic Republic of the Congo, Guatemala, Guyana, Peru, and Greece.

The surge in fire-related forest disturbance accounts for the total forest disturbance area increase. The average annual area of forest disturbance due to fire increased 2.2 times between 2002–2022 and 2023–2024 intervals, while the average annual area of forest disturbance due to other proximate causes increased only 1.2 times (Fig. 1*B*). The percentage of fire-related forest disturbance of total disturbance area was the highest in boreal forests, where it increased from 73 to 86% between the 2002–2022 and 2023–2024 intervals. While the share of fire in total forest disturbance area in tropical rainforest was small, it increased considerably from 8 to 19% between the 2002–2022 and 2023–2024 intervals.

We observed a dramatic increase in disturbance due to fire within forests with high aboveground biomass density and conservation value. An Intact Forest Landscape (IFL) is a mosaic of forest and naturally treeless ecosystems with no remotely detected signs of human activity and a minimum area of 50,000 ha (5). We observed a sharp increase in forest disturbance due to fire within IFL during the last decade. The average annual fire-related disturbance in 2023 and 2024 was 2.5 times higher than the average for the 2002–2022 interval (Fig. 1*C*). The area of annual forest disturbance due to fire in IFL has a statistically significant increasing trend (Mann–Kendall test, *P* < 0.05).

**Fig. 2.**
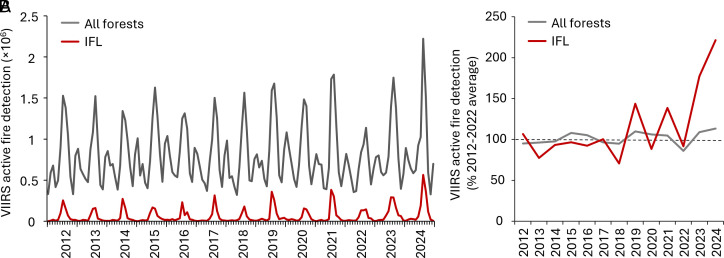
(*A*) VIIRS monthly active fire detection number within all forests and IFL, 2012 to 2024. (*B*) VIIRS annual active fire detection number as a percentage of the 2012 to 2022 average for all forests and IFL, 2012 to 2024.

Changes in monthly and annual active fire detections from the Visible Infrared Imaging Radiometer Suite (VIIRS) from 2012 to 2024 confirm the observed changes in forest disturbance due to fire (Fig. 2*A*). The number of active fire detections in August 2024 was 25% higher than the monthly maximum value since 2012. The annual fire detection number was the highest in 2024; compared to the average 2012 to 2022, it was 14% higher for all forests and 121% higher for IFL (Fig. 2*B*). We found a statistically significant increasing trend in the monthly number of active fire detections within IFL (Seasonal Mann–Kendall test, *P* < 0.05).

## Discussion

Ongoing human-caused climate change most likely drove the observed increase in forest disturbance due to fire. Climate change is responsible for increases in fire season length, fuel dryness, and frequency of fire weather events (6, 7). The years 2023 and 2024 were warmest globally since 1850 (8) contributing to the observed increase in the annual area of forest disturbance due to fire in most climate domains and continents. Agricultural land use conversion and forest fragmentation by logging, mining, and transportation infrastructure increased the probability of fire ignition, both within the boreal (9) and tropical (10) forests. Traditional agriculture that relies on fire use, such as shifting cultivation, was responsible for escaped fires due to changes in fire seasons and fuel moisture outside the historical range (11). We suggest that in humid tropical forests, the observed increase in fire-related forest disturbance during 2016 and 2024 was led by increased fire ignition from land use and infrastructure, coupled with the higher extremity of fire weather related to the warm phase of El Niño Southern Oscillation (ENSO). The 2024 extreme heat and drought in South America and Africa (8) enabled the spread of accidental, intentional, and naturally ignited fires in tropical forests.

The IFL include the least-disturbed natural forests with high canopy cover, tree height, and aboveground biomass density. Tropical IFL have 2.7 times higher aboveground biomass than degraded and fragmented forest landscapes (5). The observed increase in their annual reduction due to logging, land use conversion, and fires resulted in a disproportionately high risk of biodiversity loss, carbon emissions, and reduction of other services. The recent increase in forest fire emissions (12) is a consequence of the observed increase in forest disturbance due to fire within high aboveground biomass density forests, such as tropical IFL.

Climate models predict the future increase in frequency and intensity of fire weather and the increase in forest fires (7). The increase in area, frequency, and severity of forest fires has a positive feedback to further climate warming due to CO_2_ emissions (12). Reducing CO_2_ emissions from all anthropogenic sources is considered a primary goal in preventing predicted forest ecosystem transformation (13). Given that the expansion of road infrastructure, selective logging, and land use conversion within pristine forests are important drivers of fire regime intensification (5, 10), establishing and enhancing protection of the remaining primary and intact forests is another important solution to reduce fire danger. Finally, national and local programs for fire management, fire prevention, and public education are critical to prevent catastrophic forest fires, but for most regions, they are poorly developed and lack financial support (14).

## Materials and Methods

The global annual gross forest loss and gross forest loss due to fire datasets are produced by the Global Land Analysis and Discovery Lab at the University of Maryland with support from the World Resources Institute. The monitoring methodology relies on the global Landsat data archive (1, 3). Our forest definition includes all natural, managed, or planted trees. Forest disturbance was defined as a gross loss of more than half of the tree canopy cover within the satellite data pixel footprint (approximately 30 × 30 m on the Equator). Our forest disturbance definition does not discriminate between temporary tree canopy removal and deforestation. Our fire definition includes wildfires, escaped fires, and intentionally set fires; it excludes the burning of previously felled trees, such as those within shifting cultivation (3). The 2001 to 2024 dataset is publicly available online (2, 15). The VIIRS active fire products are available from the NASA EOSDIS Land Processes Distributed Active Archive Center. The analysis regions are identical to the regions used in ref. 3. Detailed information on the methods is provided in the *SI Appendix*.

## Supplementary Material

Appendix 01 (PDF)

## Data Availability

Global forest change data is available from https://glad.earthengine.app/ (2, 15).
