# Supplementary material for: Unprecedentedly high global forest disturbance due to fire in 2023 and 2024
Source: Proc Natl Acad Sci U S A. 2025 Jul 21;122(30):e2505418122. doi: 10.1073/pnas.2505418122 (PMC12318170; doi:10.1073/pnas.2505418122)
Supplement: Supplementary file 1 — Appendix 01 (PDF) [file pnas.2505418122.sapp.pdf]

PNAS

Supporting Information for
Unprecedentedly high global forest disturbance due to fire in 2023 and 2024

Potapov P., Tyukavina A., Turubanova S., Hansen M.C., Giglio L., Hernandez-Serna A., Lima A., Harris N., Stolle F.

Potapov P., Hansen M.C.
Email: potapov@umd.edu, mhansen@umd.edu

This PDF file includes:

- Extended Methods
- SI References

Extended Methods

Forest Disturbance and Forest Disturbance due to Fire

The global forest gross loss product was developed by (1) and updated annually using the method developed by (2). The annual change detection method relies on the Landsat Analysis Ready Data 16-day time series product converted to annual features that facilitate mapping forest change globally using a regionally calibrated machine learning tool (2, 3). We define forest as woody vegetation with a canopy height of ≥5 m. Our forest definition includes all natural, managed, or planted trees. Forest disturbance was defined as a gross loss of more than half of the tree canopy cover within the Landsat data pixel footprint (approximately 30×30 m on the Equator) (1). Our forest disturbance product is sensitive to substantial reduction of tree canopy within a Landsat pixel (e.g., tree canopy removal within ≥50% of the Landsat pixel) but not sensitive to low-intensity partial disturbances and temporary defoliation events, such as low-intensity and understory forest fires. Our forest disturbance definition does not discriminate between temporary tree canopy removal and deforestation. The annual forest disturbance product represents the first year of disturbance detection since 2001. We do not show the repeated forest disturbance events after the year 2001.

The global forest disturbance product validation at the global (2001-2012, (1)) and regional (2001-2017, (2)) scales yielded a high overall accuracy of 99% and 93%, respectively. We updated the product for the years 2023 and 2024 using the same source Landsat data and change detection models that we have used globally since 2015 (4).

The global forest disturbance due to fire is a subset of the global forest disturbance dataset (5). The fire attribution of the mapped forest gross loss was performed using a machine learning tool calibrated manually to identify fire-related disturbances using the annual features derived from the Landsat Analysis Ready Data 16-day time series (3). Each forest disturbance event (Landsat pixel) was attributed as a disturbance due to fire or other proximate causes (6). Our fire definition includes wildfires, escaped fires from slash-and-burn agriculture, hunting, and other human activities, and intentionally set fires. Burning of previously felled trees, such as soil preparation within the slash-and-burn agriculture cycle, is excluded from our definition of forest disturbance due to fire (5). Like the global forest disturbance data, we do not show repeated fires or fires that happened after the forest clearing event detected earlier.

We validated the global forest disturbance due to fire data product globally for the 2001-2019 interval (5). The global user's and producer's accuracies of the forest disturbance due to fire exceeded 80%. A sample-based analysis showed that the product slightly underestimated the forest disturbance due to fire by about 8% globally, which means that the estimated area of burned forests may be higher. We have also validated map-based forest disturbance due to fire trends at the continental and global scales via comparison with sample-based trends. The same source data and fire-related disturbance attribution model were implemented for the product updates in 2021-2024.

Collecting and Analyzing Regional Statistics

The global forest disturbance and forest disturbance due to fire data were collected using pixel counts and pixel areas from the existing global datasets (4, 6). We used four sets of regions to extract global statistics (5): climate domains obtained from the FAO Global Ecological Zones 2010 (7), continents and countries derived using the global administrative boundary dataset (8), and the global extent of the Intact Forest Landscapes 2000 (IFL, (9, 10)). For each year, we calculated the area of total forest disturbance and forest disturbance due to fire. We confirmed trend significance using the original Mann-Kendall test for annual data and the seasonal Mann-Kendall test for monthly data with significance levels set at $\alpha = 0.05$ and 0.10 (11).

VIIRS Active Fire Analysis

The active fire detection product is generated by NASA's Land Science Investigator Processing System using data from the Visible Infrared Imaging Radiometer Suite (VIIRS). The VIIRS sensor is currently deployed on three platforms (S-NPP, NOAA-20, and NOAA-21), which were launched in 2011, 2017, and 2022, respectively. Each VIIRS instrument provides global data coverage every 12 hours (and more frequently at high latitudes). The active fire detection algorithm primarily uses middle- and thermal-infrared imagery data at 375-m spatial resolution (12). The 375-m VIIRS active fire product is generated for each VIIRS sensor separately.
Here, we employed the monthly S-NPP VIIRS active fire detection product (VNP14IMGML) generated by the monthly integration of all fire detection events from the S-NPP VIIRS since late January 2012 (13). We selected events with "nominal" and "high" confidence and "presumed vegetation fire" type to ensure that only valid active fire detections were included in the analysis.

For the analysis of monthly VIIRS active fire detections, we used the 2012 global extent of forest (the first year of the VIIRS product) and the 2000 extent of IFL. To create the 2012 forest extent, we integrated the tree canopy cover product for the year 2000 (1) with the annual forest loss data from 2001 to 2011. We considered Landsat pixels with a canopy cover of ≥10% and excluded pixels where forest loss was detected from 2001 to 2011 to create the forest mask. To reduce the non-forest fire commission errors and the effect of the difference between the Landsat (30 m) and VIIRS (375 m) spatial resolution, for both analysis mask (all forests and IFL), we calculated the proportion of the target class extent within a circular window with a radius of 400 m. Only pixels with ≥0.75 proportion of the target class within a 400 m radius window were used for our analysis.

SI References

1. M. C. Hansen, *et al.*, High-resolution global maps of 21st-century forest cover change. *Science* **342**, 850–853 (2013).

2. P. Potapov, *et al.*, Annual continuous fields of woody vegetation structure in the Lower Mekong region from 2000-2017 Landsat time-series. *Remote Sensing of Environment* **232**, 111278 (2019).

3. P. Potapov, *et al.*, Landsat analysis ready data for global land cover and land cover change mapping. *Remote Sensing* **12**, 426 (2020).

4. Global Forest Change 2001-2024. https://glad.earthengine.app/view/global-forest-change.

5. A. Tyukavina, *et al.*, Global trends of forest loss due to fire from 2001 to 2019. *Front. Remote Sens.* **3** (2022).

6. Global Forest Loss due to Fire 2001-2024. https://glad.earthengine.app/view/global-forest-loss-due-to-fire.

7. FAO, *Global ecological zones for FAO forest reporting: 2010 Update* (Rome, Italy: FAO, 2012).

8. Database of Global Administrative Areas (GADM). https://gadm.org/.

9. P. Potapov, *et al.*, The last frontiers of wilderness: Tracking loss of intact forest landscapes from 2000 to 2013. *Science Advances* **3**, e1600821–e1600821 (2017).

10. Intact Forest Landscapes 2000. https://intactforests.org/.

11. M. M. Hussain, I. Mahmud, pyMannKendall: a python package for non parametric Mann Kendall family of trend tests. *Journal of Open Source Software* **4**, 1556 (2019).

12. W. Schroeder, P. Oliva, L. Giglio, I. a. Csiszar, The New VIIRS 375m active fire detection data product: Algorithm description and initial assessment. *Remote Sensing of Environment* **143**, 85–96 (2014).

13. W. Schroeder, L. Giglio, J. Hall, *Collection 2 Visible Infrared Imaging Radiometer Suite (VIIRS) 375-m Active Fire Product User's Guide Version 1.* (NASA, 2024).
